# Impact of cyclosporine A-related single nucleotide polymorphisms on post-transplant outcomes in pediatric hematologic malignancy patients undergoing allogeneic hematopoietic stem cell transplantation

**DOI:** 10.3389/fimmu.2025.1615976

**Published:** 2025-07-22

**Authors:** Qi Ji, Senlin Zhang, Minyuan Liu, Weiliang Zhang, Lixia Liu, Yutan Chai, Li Gao, Bohan Li, Zhizhuo Du, Yixin Hu, Peifang Xiao, Jing Ling, Liyan Fan, Xinni Bian, Hong Chen, Jie Li, Jun Lu, Yongping Zhang, Shuiyan Wu, Jiayue Qin, Shaoyan Hu, Yizhen Li

**Affiliations:** ^1^ Department of Hematology and Oncology, Children’s Hospital of Soochow University, Suzhou, China; ^2^ Department of Medical Affairs, Acornmed Biotechnology Co., Ltd., Beijing, China; ^3^ Department of Pediatric Hematology and Oncology, Key Laboratory of Higher Education Institutions in Jiangsu Province, Suzhou, China; ^4^ Department of Pediatric Intensive Care Unit, Children’s Hospital of Soochow University, Suzhou, China

**Keywords:** HSCT, CSA, SNP, complication, prognosis

## Abstract

**Background:**

Calcineurin inhibitors (CNIs), such as cyclosporine A (CsA), are widely used as immunosuppressants for both prophylactic and therapeutic purposes in patients with graft-versus-host disease (GVHD) after allogeneic hematopoietic stem cell transplantation (allo-HSCT). CsA-related transporters and metabolic enzymes single nucleotide polymorphisms (SNPs) are associated with the efficacy of CsA in individuals. However, few studies have explored how CsA-related SNPs correlate with post-transplant complications and prognosis.

**Methods:**

Here, our study involved 128 pediatric hematological malignancy patients undergoing allo-HSCT with GVHD prophylaxis based on CsA. All patients were detected for CsA-related SNPs. We investigated the associations between the CsA-related SNPs and post-transplant complications and prognosis.

**Results:**

We examined twenty-three CsA-related SNPs. Based on multivariate analysis using Cox regression, we identified umbilical cord blood HSCT and donor-recipient HLA matches of 9/10-10/10 as independent factors for peri-engraftment syndrome (hazard ratio (HR) = 2.82, *P* = 0.008; HR = 0.30, *P* = 0.021, respectively); recipient weight ≤ 26 kg, donor-recipient major or minor ABO blood type mismatch, and *CYP2C19* (99T>C) variant genotype as independent risk factors for grades II-IV acute GVHD (aGVHD) (HR = 2.08, *P* = 0.008; HR = 2.56, *P* = 0.008; HR = 2.22, *P* = 0.014; HR = 1.80, *P* = 0.042, respectively); matched unrelated donor HSCT and donor-recipient HLA matches of 9/10-10/10 as independent factors for Epstein-Barr virus infection (HR = 5.22, *P* = 0.019; HR = 0.13, *P* = 0.003); *CYP3A5* (219-237C>T) variant genotype as an independent protective factor for cytomegalovirus infection (HR = 0.58, *P* = 0.025); recipient being male, age at transplantation ≤ 104 months, *ABCB1* (1236C>T) CT/TT genotype, and *SLCO1B1* (1865 + 4846T>C) TC/CC genotype as independent factors for hemorrhagic cystitis (HR = 2.65, *P* = 0.024; HR = 0.46, *P* = 0.023; HR = 0.39, *P* = 0.030; HR = 0.32, *P* = 0.001, respectively); and donor-recipient HLA matches of 9/10-10/10 as an independent protective factor for capillary leak syndrome (CLS) (HR = 0.19, *P* = 0.031). Additionally, we found a body weight ≤ 26 kg, CLS after HSCT, *SLC29A1* (-162 + 228A>C) AC/CC genotype were independent factors for both disease-free survival (HR = 0.38, *P* = 0.022; HR = 2.64, *P* = 0.023; HR = 0.29, *P* = 0.016, respectively) and overall survival (HR = 0.27, *P* = 0.007; HR = 3.83, *P* = 0.003; HR = 0.22, *P* = 0.005, respectively).

**Conclusion:**

Our study revealed correlations between CsA-related transporters and metabolic enzymes SNPs and post-transplant complications and prognosis, contributing to a better understanding of the interindividual difference in efficacy. Future studies on adjusting the dosage of drugs based on SNPs in clinical practice may be one of the options for improving the HSCT outcomes.

## 1 Introduction

Allogeneic hematopoietic stem cell transplantation (allo-HSCT) has emerged as an effective treatment for refractory or relapsed hematological malignancies, including leukemia, lymphoma and myelodysplastic syndrome (1). Despite its therapeutic benefits, graft-versus-host disease (GVHD) remains one of the most significant complications following HSCT and a leading cause of transplant-related morbidity and mortality. The prognosis for patients with severe (grade III-IV) GVHD remains particularly poor, with reported survival rates varying from 25% in the adult population to 55% in pediatric cohorts (2). While the treatment with immunosuppressants has reduced the incidence and mortality of GVHD, this has been accompanied by an increased risk of other clinically significant complications, including opportunistic infections (particularly cytomegalovirus (CMV) and Epstein-Barr virus (EBV) reactivation), hemorrhagic cystitis (HC), and capillary leak syndrome (CLS) (3).

Calcineurin inhibitors (CNIs), like cyclosporine A (CsA), remain a cornerstone in both the prophylaxis and treat GVHD after HSCT, which significantly enhanced the overall survival (OS) rates after transplantation (4). Cyclosporine A (CsA) is an 11-amino acid cyclic peptide obtained from fungal fermentation. CsA works by selectively inhibiting the activity of calcineurin, which suppresses the lymphocyte activation and proliferation, leading to its immunosuppressive effects (5). The early post-transplant concentration of CsA is closely associated with GVHD risk, highlighting the importance of individualized drug management (6).

CsA is mainly metabolized by enzymes from the CYP450 family, predominantly CYP3A4 and CYP3A5 (7). For example, CYP3A5*3C, a splicing variant in intron 3, leads to aberrant mRNA splicing and a premature stop codon, resulting in a truncated, non-functional protein (8). Several studies have demonstrated that the CYP3A5*3C/*3C genotype correlates with a higher CsA C_0_/dose in early renal transplant patients (9, 10). Moreover, CYP3A4*1G and CYP3A5*3C are in strong linkage disequilibrium in Asians, and individuals carrying the CYP3A5*1 allele are more likely to co-inherit the CYP3A4*1G allele than the CYP3A4*1 allele. These indicate differences in individual single nucleotide polymorphism (SNP) are the primary factors affecting the metabolic activities of these enzymes.

In addition to metabolic enzymes, CsA acts as a substrate for various influx and efflux transporters, such as ABCB1 and SLCO1B1 (11, 12). SNP variations in transporter coding gene, such as *ABCB1*, have been associated with altered CsA pharmacokinetics. Notably, *ABCB1* 3435CC genotype carriers tend to require higher CsA doses than 3435TT carriers, particularly in Asian populations and during the early post-transplant period (13). Other metabolic pathways, including UGT1A8 and BMP-related pathways, may also contribute to CsA disposition, although these associations remain less well defined (14, 15).

However, limited reports have examined the relationship between gene SNPs and clinical complications after transplantation.Our study aimed to identity gene SNPs of CsA-related transporters and metabolic enzymes, including ABC, BMP, CYP, MTHFR, MTRR, SLC, STIM1, UGT1A8 family, and correlate these SNPs with common post-transplant clinical complications in 128 pediatric hematological malignancy patients.

## 2 Materials and methods

### 2.1 Study designs

We performed a retrospective cohort study of 128 patients who received allo-HSCT in Children’s Hospital of Soochow University (Suzhou, China) between June 2019 and June 2021 (**Table 1**). The Institutional Review Board at Children’s Hospital of Soochow University approved the study (No. 2023CS105) and all recipients and donors provided written informed consent according to the Declaration of Helsinki. The study inclusion criteria were as follows: (1) allo-HSCT; (2) first-time transplant; (3) patients aged less than 18 years old; (4) received CsA-based GVHD prophylaxis; (5) presence of informed consent to CsA-related genotyping. Patients were excluded if they: (1) discontinued CsA prematurely for any reason; (2) had a documented allergy or intolerance to CsA.

**Table 1:** Patient clinical characteristics.

Demographic information	Patient cohort (n = 128)
Age, median (IQR) months	104 (54, 144)
Weight, median (IQR) kg	26 (17.45, 38.5)
Neutrophil recovery day, median (IQR) day	12 (11, 13)
Platelet recovery day, median (IQR) day	11 (10, 15)
Diagnosis, n (%)	
AML	62 (48.4%)
ALL	57 (44.5%)
MAPL	5 (3.9%)
CML	1 (0.8%)
JMML	2 (1.6%)
T-LBL	1 (0.8%)
Gender, n (%)	
Female	37 (28.9%)
Male	91 (71.1%)
ABO match, n (%)	
Matched	59 (46.1%)
Major mismatched	24 (18.8%)
Minor mismatched	35 (27.3%)
Mismatched	10 (7.8%)
Donor type, n (%)	
Haplo-HSCT	84 (65.6%)
MSD-HSCT	12 (9.4%)
MUD-HSCT	12 (9.4%)
UCB-HSCT	20 (15.6%)
Transplantation, n (%)	
BM + PB + CB	40 (31.2%)
PB	49 (38.3%)
CB	13 (10.2%)
BM + PB	22 (17.2%)
BM	1 (0.8%)
BM + CB	1 (0.8%)
PB + CB	2 (1.6%)
HLA match, n (%)	
5/10	44 (34.4%)
9/10-10/10	42 (32.8%)
6/10-8/10	42 (32.8%)
BM state before transplantation, n (%)	
PR/NR	9 (7%)
CR	119 (93%)
MNC, median (IQR) ×10^8^/kg	6.825 (4.98, 8.3075)
CD34+ cell, median (IQR) ×10^6^/kg	6.585 (4.7625, 8.6775)

SNP, single nucleotide polymorphism; IQR, interquartile range; AML, acute myeloid leukemia; ALL, acute lymphoblastic leukemia; MAPL, mixed-phenotype acute leukemia; CML, chronic myeloid leukemia; JMML, juvenile myelomonocytic leukemia; T-LBL, T-cell lymphoblastic lymphoma; MNC, mononuclear cells; BM, bone marrow; CR, complete remission; PR, partial remission; NR, no remission; GVHD, graft-versus-host disease; PB, peripheral blood; CB, cord blood; HLA, human leukocyte antigen.

### 2.2 Conditioning regimen and GVHD prophylaxis

All patients in the conditioning stage received a myeloablative regimen incorporating the combination of busulfan and cyclophosphamide (BuCy). Drug dosages were tailored according to individual patient parameters including height, weight, body surface area to optimize the therapeutic regimen in terms of both scientific rationale and efficacy. Busulfan was administered intravenously at 0.8 mg/kg every 6 hours for 3 or 4 consecutive days, and cyclophosphamide was given intravenously at a total dose of 120 mg/kg over 2 or 3 days. In certain cases, supplementary regimens like fludarabine and cytarabine (FLAG) or cladribine and cytarabine (CLAG) may be introduced as bridging therapies alongside the BuCy transplantation. In the FLAG group, fludarabine (30 mg/m²/day) and cytarabine (2 g/m²/day) were administered intravenously for 4 days, with G-CSF (5 µg/kg/day) given subcutaneously for 5 days. In the CLAG group, cladribine (5 mg/m²/day) and cytarabine (2 g/m²/day) were administered intravenously for 3 or 5 days, along with subcutaneous granulocyte-colony stimulating factor (G-CSF, 5 µg/kg/day) for 4 or 6 days.

The GVHD prophylaxis included oral CsA and MMF. CsA was administered at 6–10 mg/kg/day every 12 hours. MMF was given at 15 mg/kg/day every 12 hours, with the dose halved on day 30 post-transplant and discontinued by day 45. Umbilical cord blood (UCB) transplantation and fully matched sibling transplantation typically require CsA levels of approximately 150–200 ng/ml, while unrelated fully matched transplantation and haploidentical transplantation require CsA levels of around 200–250 ng/ml. Additionally, some patients concurrently received oral methotrexate (MTX) as a prophylactic measure against GVHD, administered at 15 mg/m² on day +1 and 10 mg/m² on days +3, +6, and +11.

### 2.3 Post-transplant evaluation

Neutrophil engraftment was defined as achieving an absolute neutrophil count ≥ 0.5×10^9^/L for three consecutive days. Platelet engraftment was defined as reaching a platelet count ≥ 20×10^9^/L without requiring transfusion support for seven consecutive days. A myeloablative conditioning regimen, defined as Bu exceeding 8 mg/kg. Primary graft failure (GF) was defined as the failure of neutrophil engraftment at day +28 post-HSCT, secondary GF was defined as the development of neutrophil count < 0.5×10^9^/L occurring after the initial engraftment. The diagnosis and grading of aGVHD and chronic GVHD (cGVHD) were performed by the transplant center using standard criteria (16, 17). The definition of hemorrhagic cystitis (HC), capillary leak syndrome (CLS) and Peri-engraftment syndrome (Peri-ES) follow as per previous standards (18–20). CMV and EBV viremia was defined by a viral DNA level ≥ 500 copies/mL in plasma.

### 2.4 Next-generation sequencing experiments

Genomic DNA was isolated from bone marrow diagnostic samples. A total of 27 genes was used for next-generation sequencing (NGS) at Acornmed Biotechnology Co., Ltd (Tianjin, China). The rest standards were same as our previous study (21). Based on literature review (13, 22–27), a total of 23 SNPs of CsA-related transporters and metabolic enzymes were analyzed in our cohort (**Table 2**).

**Table 2:** CsA-related transporters and metabolic enzymes SNP frequencies.

Gene family	SNP	Genotype	Patient, n (%)	Theoretical frequency, n	*P*-value
*ABC*	*ABCB1* (1000-44C>T)	CC	54 (42.2%)	55.13	0.66
		CT	60 (46.9%)	57.75	
		TT	14 (10.9%)	15.13	
	*ABCB1* (1199C>T)	CC	128(%)	128	NA
		CT	0 (%)	0	
		TT	0 (%)	0	
	*ABCB1* (1236C>T)	CC	14 (10.9%)	15.13	0.66
		CT	60 (46.9%)	57.75	
		TT	54 (42.2%)	55.13	
	*ABCB1* (1554 + 24A>G)	AA	54 (42.2%)	55.13	0.66
		AG	60 (46.9%)	57.75	
		GG	14 (10.9%)	15.13	
	*ABCB1* (1725 + 38C>T)	CC	54 (42.2%)	55.13	0.66
		CT	60 (46.9%)	57.75	
		TT	14 (10.9%)	15.13	
	*ABCB1* (3435C>T)	CC	47 (36.7%)	45.13	0.49
		CT	58 (45.3%)	61.75	
		TT	23 (18%)	21.13	
*BMP*	*BMP7* (g.57126159C>T)	CC	118 (92.2%)	118.20	0.65
		CT	10 (7.8%)	9.61	
		TT	0 (%)	0.20	
*CYP*	*CYP2C19* (-3402C>T)	CC	127 (99.2%)	127.00	0.96
		CT	1 (0.8%)	1.00	
		TT	0 (%)	0.00	
	*CYP2C19* (636G>A)	GG	115 (89.8%)	115.33	0.55
		GA	13 (10.2%)	12.34	
		AA	0 (%)	0.33	
	*CYP2C19* (681G>A)	GG	50 (39.1%)	55.13	0.04
		GA	68 (53.1%)	57.75	
		AA	10 (7.8%)	15.13	
	*CYP2C19* (-806C>T)	CC	127 (99.2%)	127.00	0.96
		CT	1 (0.8%)	1.00	
		TT	0 (%)	0.00	
	*CYP2C19* (991A>G)	AA	0 (0%)	0.50	0.45
		GA	16 (12.5%)	15.00	
		GG	112 (87.5%)	112.50	
	*CYP2C19* (99T>C)	TT	99 (77.3%)	100.64	0.15
		TC	29 (22.7%)	25.71	
		CC	0 (0%)	1.64	
	*CYP2C8* (1291 + 106T>C)	TT	16 (12.5%)	19.14	0.24
		CT	67 (52.3%)	60.71	
		CC	45 (35.2%)	48.14	
	*CYP3A4* (-392C>T)	CC	0 (0%)	0	NA
		CT	0 (0%)	0	
		TT	128(%)	128	
	*CYP3A5* (219-237C>T)	CC	68 (53.1%)	69.77	0.42
		CT	53 (41.4%)	49.46	
		TT	7 (5.5%)	8.77	
*MTHFR*	*MTHFR* (665C>T)	CC	29 (22.7%)	25.83	0.26
		CT	57 (44.5%)	63.34	
		TT	42 (32.8%)	38.83	
*MTRR*	*MTRR* (66A>G)	AA	79 (61.7%)	77.35	0.40
		AG	41 (32%)	44.31	
		GG	8 (6.2%)	6.35	
*SLC*	*SLC29A1* (-162 + 228A>C)	AA	10 (7.8%)	10.70	0.76
		AC	54 (42.2%)	52.61	
		CC	64 (50%)	64.70	
	*SLCO1B*1 (1865 + 4846T>C)	TT	51 (39.8%)	49.38	0.54
		TC	57 (44.5%)	60.25	
		CC	20 (15.6%)	18.38	
	*SLCO1B1* (521T>C)	TT	106 (82.8%)	104.22	0.07
		TC	19 (14.8%)	22.56	
		CC	3 (2.3%)	1.22	
*STIM1*	*STIM1* (1138-52A>C)	AA	0 (0%)	0.33	0.55
		AC	13 (10.2%)	12.34	
		CC	115 (89.8%)	115.33	
*UGT1A8*	*UGT1A8* (518C>G)	CC	32 (25%)	35.07	0.28
		CG	70 (54.7%)	63.86	
		GG	26 (20.3%)	29.07	

SNP, single nucleotide polymorphism; NA, not applicable.

### 2.5 Statistical analysis

All data analyses were performed using SPSS software (version 22.0) or R (version 3.5.2). Differences between two groups were compared using independent sample t-tests or Mann-Whitney U non-parametric tests for quantitative variables. For categorical variables, Chi-square tests or Fisher’s exact test were employed. Factors with a *P*-value < 0.2 via univariate analysis using the Log-rank test were further included in a Cox regression model for multivariate analysis of factors related to clinical outcome events. Genetic conformity to Hardy-Weinberg equilibrium was analyzed using the Chi-square test, with *P* > 0.05 considered to indicate adherence to Hardy-Weinberg equilibrium. All tests were two-sided, and statistical significance was set at *P* < 0.05.

## 3 Results

### 3.1 Patient characteristics

This study involved 128 pediatric patients with hematologic malignancies who underwent HSCT, with their clinical characteristics detailed in **Table 1**. The median age at HSCT was 104 months. The most common disease types included acute lymphoblastic leukemia (ALL, 44.5%), acute myeloid leukemia (AML, 48.4%), mixed phenotype acute leukemia (MAPL, 3.9%), and juvenile myelomonocytic leukemia (JMML, 1.6%). All patients received a prophylaxis regimen for GVHD based on CsA. Ultimately, all patients achieved hematopoietic recovery. The median time for neutrophil and platelet engraftment were 12 days (interquartile range (IQR), 11-13) and 11 days (IQR, 10-15), respectively.

### 3.2 CsA-related transporters and metabolic enzymes SNP

**Figure 1:**
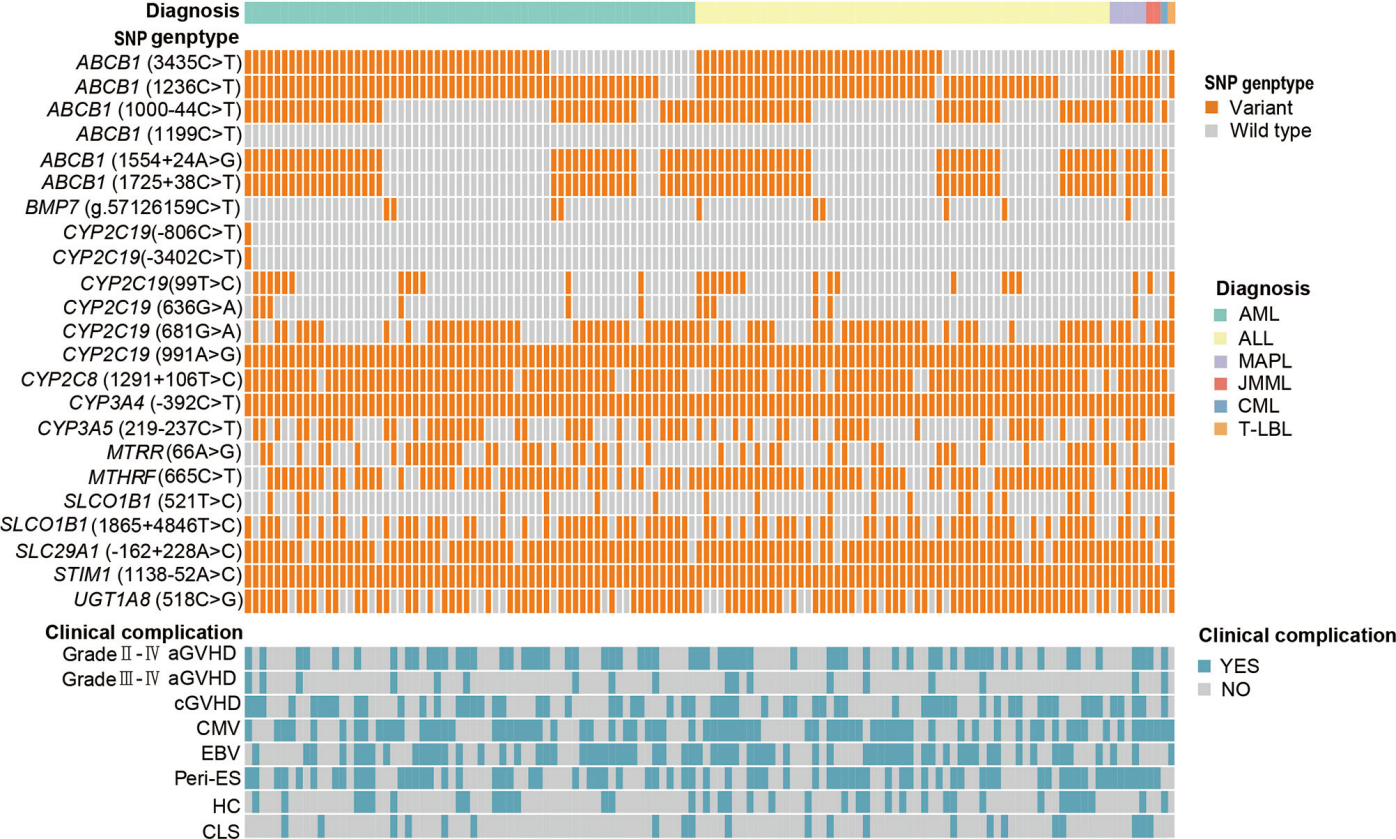
CsA-related transporters and metabolic enzymes gene SNP frequencies in 128 pediatric patients receiving allo-HSCT. Heatmap shows the specific variants in each patient based on different genotypes, according to different subtypes of malignant hematological diseases. CsA, cyclosporine A; SNP, single nucleotide polymorphism; allo-HSCT, allogeneic hematopoietic stem cell transplantation; AML, acute myeloid leukemia; ALL, acute lymphoblastic leukemia; MAPL, mixed-phenotype acute leukemia; CML, chronic myeloid leukemia; JMML, juvenile myelomonocytic leukemia; T-LBL, T-cell lymphoblastic lymphoma. aGVHD, acute graft-versus-host disease; cGVHD, chronic GVHD; CMV, cytomegalovirus; EBV, Epstein-Barr virus; Peri-ES, peri-engraftment syndrome; HC, hemorrhagic cystitis; CLS, capillary leak syndrome.

**Figure 1** and **Table 2** provide descriptions of SNPs related to CsA-related transporters and metabolic enzymes. We examined twenty-three CsA-related SNPs. The most frequently observed genotypes were *SLC29A1* (-162 + 228A>C), followed by *ABCB1* (1236C>T) and *CYP2C8* (1291 + 106T>C) (**Figure 1**). Among the CsA-related SNPs, *CYP2C19* (681G>A), *ABCB1* (1199C>T), and *CYP3A4* (-392C>T) did not conform to Hardy-Weinberg equilibrium, whereas the remaining SNPs did (*P* > 0.05, **Table 2**).

### 3.3 Correlation between CsA-related SNPs and post-transplant complications

We examined how CsA-related transporters and metabolic enzymes SNPs are associated with post-transplant complications, including GVHD, viral infection, Peri-ES, HC and CLS. This study involved 128 patients, of whom 61 (47.7%) had grades II-IV acute GVHD (aGVHD), and 18 (14.1%) experienced grades III-IV aGVHD. Additionally, 61 patients (47.7%) had cGVHD, 74 (57.8%) had CMV infection, 68 (53.1%) had EBV infection, 71 (55.5%) had Peri-ES, 38 (29.7%) had HC, and 21 (16.4%) had CLS.

We performed univariate analysis with the Log-rank test, and found that the recipients with major ABO blood type mismatches had a significantly higher incidence of Peri-ES than those with fully matched cases (*P* = 0.043). Moreover, recipients of HLA-matched sibling donor HSCT (MSD-HSCT) and matched unrelated donor HSCT (MUD-HSCT) experienced lower incidences of Peri-ES compared to those undergoing recipients of HLA-matched sibling donor HSCT (MSD-HSCT) and matched unrelated donor HSCT (MUD-HSCT) experienced lower incidences of Peri-ES compared to those undergoing haploidentical HSCT (Haplo-HSCT) (*P* = 0.014, *P* = 0.043, respectively). In contrast, recipients of umbilical cord blood HSCT (UCB-HSCT) had a significantly higher incidence of Peri-ES than those receiving Haplo-HSCT (*P* = 0.049). Additionally, the incidence of Peri-ES was significantly lower in recipients with HLA matches of 9/10-10/10 compared to those with 5/10 match (*P* < 0.001). Furthermore, patients with *BMP7* (g.57126159C>T) CT/TT genotypes showed an increased incidence of Peri-ES (HR = 0.28, *P* = 0.045) (**Supplementary Table S1**). Regarding GVHD, we found that recipients with weight ≤ 26 kg had a significantly higher incidence of grades II-IV aGVHD compared to those with weight > 26kg (*P* = 0.014). UCB-HSCT recipients exhibited a higher incidence of grades II-IV aGVHD compared to those receiving Haplo-HSCT (*P* = 0.035) (**Supplementary Table S2**). Recipients with donor-recipient major ABO blood type mismatches had a significantly higher incidence of grades III-IV aGVHD compared to those with fully matched cases (*P* = 0.040) (**Supplementary Table S3**). MUD-HSCT recipients exhibited a significantly lower incidence of cGVHD than those receiving Haplo-HSCT (*P* < 0.001). Recipients with HLA matching of 9/10-10/10 had a significantly lower incidence of cGVHD than those with HLA matching of 5/10 (*P* = 0.002). Moreover, recipients with lower mononuclear nuclear cell (MNC) infusion dose (≤ 6.83×10^8^/kg) had a significantly lower incidence of cGVHD than those with a higher dose (*P* = 0.005). Carriers of the *SLCO1B1* (521T>C) TT genotype had a higher incidence of cGVHD compared to carriers of the TC/CC genotype (*P* = 0.005) (**Supplementary Table S4**). The incidence of EBV infection was significantly lower in UCB-HSCT recipients compared to Haplo-HSCT recipients (*P* = 0.010). Recipients with HLA matches of 9/10-10/10 showed a lower incidence compared to those with 5/10 HLA matches (*P* = 0.018). Wild-type alleles of *ABCB1* (1000-44C>T), *ABCB1* (1554 + 24A>G), *ABCB1* (1725 + 38C>T), and *CYP3A5* (219-237C>T) are associated with a significantly higher incidence of EBV infection compared to those with variant alleles (all *P* < 0.050). (**Supplementary Table S5**). MSD-HSCT recipients experienced significantly fewer CMV infections than those who received Haplo-HSCT (*P* = 0.046). Recipients with donor HLA matches of 9/10-10/10 had a significantly lower incidence of CMV infection compared to those with HLA matches of 5/10 (*P* = 0.006). Patients with *CYP2C8* (1291 + 106T>C) TT genotypes exhibited a significantly higher incidence of CMV infection than those carrying the variant alleles (*P* = 0.015) (**Supplementary Table S6**). Age at transplantation > 104 months and recipient weight ≥ 26 kg both increased incidence of HC (*P* = 0.040, *P* = 0.047, respectively). Recipients with donor HLA matches of 9/10-10/10 had a significantly lower incidence of HC than those with HLA matches of 5/10 (*P* = 0.014). Patients with *ABCB1* (1236C>T) CC genotype and *SLCO1B1* (1865 + 4846T>C) TT genotype showed a significantly higher incidence of HC compared to carriers with the variant alleles (*P* = 0.047, *P* = 0.007, respectively) (**Supplementary Table S7**). Recipients with donor HLA matches of 9/10-10/10 had a significantly lower incidence of CLS compared to those with HLA matches of 5/10 (*P* = 0.016) (**Supplementary Table S8**).

We constructed multivariate Cox regression analysis using variables selected by univariate Log-rank test analysis with *P* < 0.2, and the results are presented in **Supplementary Table S1**-**S8**. We identified UCB-HSCT was an independent risk factor for peri-ES (*P* = 0.008), while donor-recipient HLA matches of 9/10-10/10 was an independent protective factor for peri-ES (*P* = 0.021) (**Supplementary Table S1**). Recipients with weight ≤ 26 kg, major or minor ABO blood type mismatches, and *CYP2C19* (99T>C) TC/CC genotype were all independent risk factors for grades II-IV aGVHD (all *P* < 0.050) (**Supplementary Table S2**). MUD-HSCT increased the risk of EBV infection (*P* = 0.019), while donor-recipient HLA matches of 9/10-10/10 served as an independent protective factor for EBV infection (*P* = 0.003) (**Supplementary Table S5**). *CYP3A5* (219-237C>T) CT/TT acted independently to protect against CMV infection (*P* = 0.025) (**Supplementary Table S6**). The risk factors for HC included recipient being male, age at transplantation > 104 months, *ABCB1* (1236C>T) CC genotype, and *SLCO1B1* (1865 + 4846T>C) TT genotype (**Supplementary Table S7**). HLA matches between donors and recipients, particularly 9/10-10/10, were an independent protective factor for CLS (*P* = 0.031) (**Supplementary Table S8**).

### 3.4 Correlation between CsA-related SNPs and post-transplant prognosis

In this study, we evaluated the DFS and OS of the patients in our cohort, founding that the 5-year DFS was 78.8% ± 3.6%, and the 5-year OS was 81.9% ± 3.4%. We examined the factors that might influence patient prognosis, including DFS and OS, using both univariate Log-rank test and multivariate Cox regression analyses.

**Figure 2:**
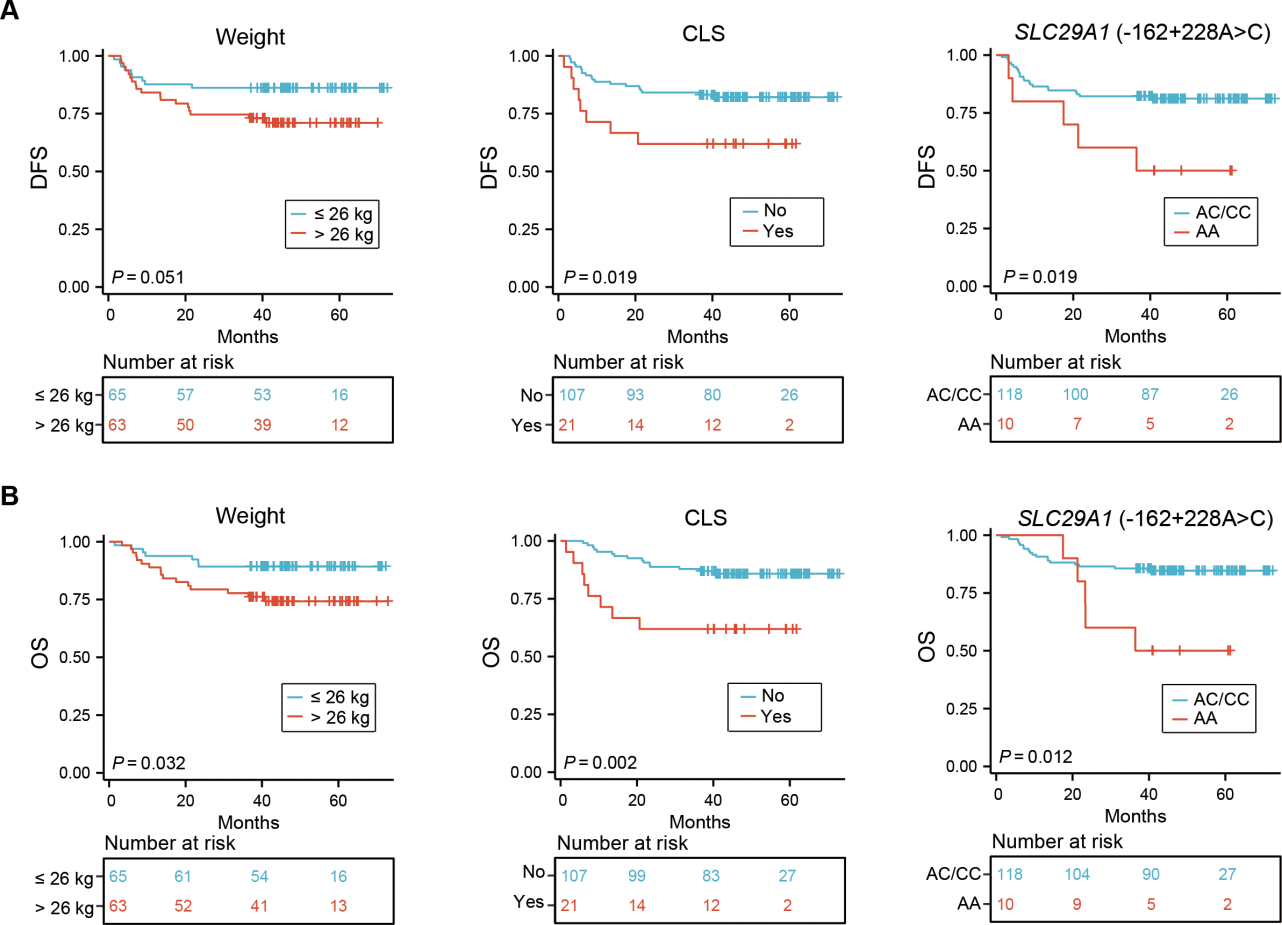
Impact of weight, CLS, and CsA-related SNP on DFS **(A)** and OS **(B)** in 128 pediatric patients receiving allo-HSCT. CLS, capillary leak syndrome; CsA, cyclosporine A; SNP, single nucleotide polymorphism; DFS, disease-free survival; OS, overall survival; allo-HSCT, allogeneic hematopoietic stem cell transplantation.

Our univariate analysis revealed that CLS and *SLC29A1* (-162 + 228A>C) AA genotype were both associated with poor prognosis in DFS (*P* = 0.019, *P* = 0.019, respectively) (**Figure 2A**, **Table 3**), and body weight ≥ 26 kg, CLS, and *SLC29A1* (-162 + 228A>C) AA genotype were identified as adverse prognostic factors in OS (*P* = 0.032, *P* = 0.002, *P* = 0.012, respectively) (**Figure 2B**, **Table 4**).

**Table 3:** Univariate and multivariate analyses of DFS.

Characteristics	Univariate analysis		Multivariate analysis	
	HR (95% CI)	*P*-value	HR (95% CI)	*P-*value
Gender, male vs. female	1.22 (0.53 - 2.77)	0.656		
Age, ≤ 104 months vs. > 104 months	0.56 (0.26 - 1.19)	0.137		
Weight, ≤ 26 kg vs. > 26 kg	0.46 (0.22 - 0.98)	0.051	0.38 (0.16 - 0.87)	0.022
ABO match				
Minor mismatched vs. Matched	0.54 (0.22 - 1.34)	0.230		
Major mismatched vs. Matched	1.02 (0.39 - 2.63)	0.974		
Mismatched vs. Matched	0.37 (0.10 - 1.44)	0.315		
Donor type				
MSD-HSCT vs. Haplo-HSCT	1.07 (0.31 - 3.70)	0.917		
MUD-HSCT vs. Haplo-HSCT	0.60 (0.22 - 1.68)	0.408		
UCB-HSCT vs. Haplo-HSCT	0.32 (0.09 - 1.13)	0.244		
HLA match				
6/10-8/10 vs. 5/10	1.06 (0.44 - 2.54)	0.905		
9/10-10/10 vs. 5/10	0.71 (0.27 - 1.83)	0.478		
BM state before HSCT, CR vs. PR/NR	0.58 (0.13 - 2.60)	0.365		
MNC, ≤ 6.83x10^8^/kg vs. > 6.83x10^8^/kg	1.21 (0.57 - 2.56)	0.629		
CD34+ cell, ≤ 6.59x10^6^/kg vs. > 6.59x10^6^/kg	1.29 (0.61 - 2.74)	0.511		
Peri-ES, yes vs. no	1.07 (0.55 - 2.51)	0.682		
II-IV aGVHD, yes vs. no	1.07 (0.50 - 2.28)	0.861		
III-IV aGVHD, yes vs. no	1.07 (0.36 - 3.19)	0.896		
cGVHD, yes vs. no	0.71 (0.33 - 1.51)	0.378		
CMV, yes vs. no	0.86 (0.40 - 1.86)	0.700		
EBV, yes vs. no	1.14 (0.53 - 2.42)	0.740		
HC, yes vs. no	1.23 (0.53 - 2.82)	0.617		
CLS, yes vs. no	2.58 (0.87 - 7.66)	0.019	2.64 (1.14 - 6.09)	0.023
SNP				
* ABCB1* (1000-44C>T) CT/TT vs. CC	1.11 (0.52 - 2.38)	0.791		
* ABCB1* (1236C>T) CT/TT vs. CC	0.67 (0.20 - 2.31)	0.464		
* ABCB1* (1554 + 24A>G) AG/GG vs. AA	1.11 (0.52 - 2.38)	0.791		
* ABCB1* (1725 + 38C>T) CT/TT vs. CC	1.11 (0.52 - 2.38)	0.791		
* ABCB1* (3435C>T) CT/TT vs. CC	1.20 (0.55 - 2.61)	0.661		
* BMP7* (g.57126159C>T) CT/TT vs. CC	0.87 (0.22 - 3.38)	0.849		
* CYP2C19* (636G>A) GA/AA vs. GG	1.06 (0.31 - 3.63)	0.923		
* CYP2C19* (99T>C) TC/CC vs. TT	0.96 (0.30 - 1.79)	0.533		
* CYP2C8* (1291 + 106T>C) TC/CC vs. TT	1.20 (0.39 - 3.68)	0.766		
* CYP3A5* (219-237C>T) CT/TT vs. CC	1.05 (0.49 - 2.23)	0.907		
* MTHFR* (665C>T) CT/TT vs. CC	1.01 (0.41 - 2.49)	0.985		
* MTRR* (66A>G) AG/GG vs. AA	0.94 (0.44 - 2.05)	0.886		
* SLC29A1* (-162 + 228A>C) AC/CC vs. AA	0.33 (0.08 - 1.45)	0.019	0.29 (0.11 - 0.79)	0.016
* SLCO1B1* (1865 + 4846T>C) TC/CC vs. TT	1.75 (0.82 - 3.75)	0.179		
* SLCO1B1* (521T>C) TC/CC vs. TT	1.49 (0.54 - 4.18)	0.382		
* UGT1A8* (518C>G) CG/GG vs. CC	1.18 (0.50 - 2.81)	0.719		

DFS, disease-free survival; HR, hazard ratio; CI, confidence interval; MSD, matched sibling donor; HSCT, hematopoietic stem cell transplantation; MUD, matched unrelated donor UCB, umbilical cord blood; HLA, human leukocyte antigen; BM, bone marrow; PR, partial remission; NR, no remission; CR, complete remission; MNC, mononuclear cells; Peri-ES, peri-engraftment syndrome; aGVHD, acute graft-versus-host disease; cGVHD, chronic GVHD; CMV, cytomegalovirus; EBV, Epstein-Barr virus; HC, hemorrhagic cystitis; CLS, capillary leak syndrome; SNP, single nucleotide polymorphism.

**Table 4:** Univariate and multivariate analyses of OS.

Characteristics	Univariate analysis		Multivariate analysis	
	HR (95% CI)	*P*-value	HR (95% CI)	*P-*value
Gender, male vs. female	1.15 (0.47 - 2.83)	0.768		
Age, ≤ 104 months vs. > 104 months	0.49 (0.21 - 1.10)	0.092		
Weight, ≤ 26 kg vs. > 26 kg	0.39 (0.17 - 0.89)	0.032	0.27 (0.10 - 0.70)	0.007
ABO match				
Minor mismatched vs. Matched	0.50 (0.19 - 1.32)	0.213		
Major mismatched vs. Matched	0.98 (0.35 - 2.75)	0.974		
Mismatched vs. Matched	0.41 (0.10 - 1.74)	0.378		
Donor type				
MSD-HSCT vs. Haplo-HSCT	0.73 (0.20 - 2.68)	0.675		
MUD-HSCT vs. Haplo-HSCT	0.70 (0.24 - 2.07)	0.562		
UCB-HSCT vs. Haplo-HSCT	0 (0 - 0)	0.089		
HLA match				
6/10-8/10 vs. 5/10	0.88 (0.34 - 2.29)	0.802		
9/10-10/10 vs. 5/10	0.67 (0.25 - 1.85)	0.450		
BM state before HSCT, CR vs. PR/NR	0.51 (0.11 - 2.51)	0.273		
MNC, ≤ 6.83x10^8^/kg vs. > 6.83x10^8^/kg	1.01 (0.44 - 2.28)	0.991		
CD34+ cell, ≤ 6.59x10^6^/kg vs. > 6.59x10^6^/kg	1.35 (0.59 - 3.05)	0.479		
Peri-ES, yes vs. no	1.26 (0.55 - 2.87)	0.586		
II-IV aGVHD, yes vs. no	0.89 (0.39 - 2.02)	0.782		
III-IV aGVHD, yes vs. no	1.39 (0.42 - 4.66)	0.547		
cGVHD, yes vs. no	0.66 (0.29 - 1.50)	0.332		
CMV, yes vs. no	0.93 (0.41 - 2.13)	0.858		
EBV, yes vs. no	1.18 (0.52 - 2.67)	0.698		
HC, yes vs. no	1.71 (0.68 - 4.27)	0.205		
CLS, yes vs. no	3.52 (1.05 - 11.81)	0.002	3.83 (1.60 - 9.19)	0.003
SNP				
* ABCB1* (1000-44C>T) CT/TT vs. CC	0.98 (0.43 - 2.23)	0.959		
* ABCB1* (1236C>T) CT/TT vs. CC	0.78 (0.20 - 2.96)	0.684		
*ABCB1* (1554 + 24A>G) AG/GG vs. AA	0.98 (0.43 - 2.23)	0.959		
*ABCB1* (1725 + 38C>T) CT/TT vs. CC	0.98 (0.43 - 2.23)	0.959		
*ABCB1* (3435C>T) CT/TT vs. CC	1.08 (0.46 - 2.53)	0.858		
*BMP7* (g.57126159C>T) CT/TT vs. CC	1.10 (0.24 - 4.94)	0.902		
*CYP2C19* (636G>A) GA/AA vs. GG	1.38 (0.35 - 5.40)	0.602		
*CYP2C19* (99T>C) TC/CC vs. TT	0.71 (0.27 - 1.88)	0.533		
*CYP2C8* (1291 + 106T>C) TC/CC vs. TT	0.98 (0.29 - 3.32)	0.971		
*CYP3A5* (219-237C>T) CT/TT vs. CC	1.55 (0.68 - 3.51)	0.297		
*MTHFR* (665C>T) CT/TT vs. CC	0.76 (0.28 - 2.08)	0.570		
*MTRR* (66A>G) AG/GG vs. AA	0.70 (0.30 - 1.62)	0.425		
*SLC29A1* (-162 + 228A>C) AC/CC vs. AA	0.30 (0.07 - 1.39)	0.012	0.22 (0.08 - 0.64)	0.005
*SLCO1B1* (1865 + 4846T>C) TC/CC vs. TT	1.28 (0.56 - 2.94)	0.571		
*SLCO1B1* (521T>C) TC/CC vs. TT	1.41 (0.47 - 4.24)	0.496		
*UGT1A8* (518C>G) CG/GG vs. CC	0.91 (0.35 - 2.35)	0.836		

OS, overall survival; HR, hazard ratio; CI, confidence interval; MSD, matched sibling donor; HSCT, hematopoietic stem cell transplantation; MUD, matched unrelated donor UCB, umbilical cord blood; HLA, human leukocyte antigen; BM, bone marrow; PR, partial remission; NR, no remission; CR, complete remission; MNC, mononuclear cells; Peri-ES, peri-engraftment syndrome; aGVHD, acute graft-versus-host disease; cGVHD, chronic GVHD; CMV, cytomegalovirus; EBV, Epstein-Barr virus; HC, hemorrhagic cystitis; CLS, capillary leak syndrome; SNP, single nucleotide polymorphism.

Multivariate analyses showed that patients with a body weight ≥ 26 kg and CLS after HSCT were independent risk factors for both DFS and OS (DFS, *P* = 0.022, *P* = 0.023; OS, *P* = 0.007, *P* = 0.003, respectively). Additionally, the study revealed that the *SLC29A1* (-162 + 228A>C) AA genotype was an independent risk factor for both DFS and OS (*P* = 0.016, *P* = 0.005, respectively) (**Tables 3**, **4**).

## 4 Discussion

Various SNPs are associated with changes in the drug metabolism and effects, explaining differences in drug responses among individuals (28, 29). SNPs affecting the metabolism of drugs like CsA, used in GVHD prophylaxis, have been related to post-HSCT complications (26).

Our study investigates the correlation between CsA-related SNPs and the transplantation outcomes in 128 patients with malignant hematological diseases patients who underwent HSCT. The incidences of various complications were as follows: grades II-IV aGVHD occurred in 47.7% of cases, grades III-IV aGVHD in 14.1%, cGVHD in 47.7%, CMV infection in 57.8%, EBV infection in 53.1%, HC in 29.7%, and CLS in 16.4%. These complication rates are consistent with previously reported data in the literature (21, 30, 31).

The multivariate analysis in this study identifies UCB-HSCT as an independent factor for peri-ES, consistent with previous reports. This observation may due to the critical role played by granulocyte-macrophage colony-stimulating factor, produced by cord blood-derived inflammatory monocytes, in driving the pathology of peri-ES in UCB transplantation (32). Also, the donor-recipient HLA matches of 9/10-10/10 have been identified as an independent protective factor for peri-ES. Previous studies indicate that mismatched donor-recipient HLA matches in allo-HSCT result in poor transplant outcomes, high incidence and severity of GVHD, slow immune reconstitution, and increased risk of severe infections (33). Some studies also suggest that peri-ES represents as an early form of aGVHD, both being immune reactions occurring post-transplant, implying a certain correlation between the occurrence of peri-ES and HLA matching (34, 35). We observed a significant decrease in CLS incidence with donor-recipient HLA matches of 9/10-10/10. Research indicates that calcineurin inhibitors, like cyclosporine and tacrolimus, used during transplantation and donor graft implantation, can activate and damage endothelial cells (36, 37). HLA matches of 9/10-10/10 can effectively reduce immunosuppressant use, decrease the GVHD incidence, endothelial cell damage and CLS incidence. Moreover, Peri-ES, CLS, and GVHD are increasingly recognized as complications driven by the release of inflammatory cytokines (38–40). It is well established that a higher degree of HLA matching results in reduced activation of donor T cells. In the early post-transplant period, donor T cells secrete pro-inflammatory cytokines such as TNF-α, IL-1, and IFN-γ, which contribute to tissue inflammation and endothelial activation (41). HLA mismatch exacerbates this cytokine storm, leading to endothelial injury. Future prospective studies are warranted to investigate the cytokine profiles and immune cell subset dynamics associated with these complications.

Regarding GVHD, the study identifies several independent risk and protective factors. This study is the first to identify independent risk factors for grades II-IV aGVHD at the time of transplantation, including recipient weight ≤ 26 kg, major and minor ABO blood type mismatched between donor and recipient, and *CYP2C19* (99T>C) TC/CC genotype. The relationship between recipient weight and GVHD remains controversial. Mehdi Yaseri et al. conducted a meta-analysis examining the relationship between obesity and post-transplant mortality and clinical outcomes in children, finding an increased risk of post-transplant mortality and aGVHD in obese children (42). Additionally, Lam T Khuat et al. suggested that obesity may increase the risk of aGVHD by affecting the microbiota composition (43). However, other studies argue that obesity has no impact on aGVHD (44). Currently, no studies have shown a correlation between low weight and high incidence of aGVHD. However, recipients with lower weight may still face GVHD-related issues, including immune reactions caused by the immunological imbalance between the graft and the host. Lower weights may result in an insufficient immune system to regulate these reactions effectively. Nevertheless, research in this area is relatively scarce, and further in-depth studies are needed to confirm this relationship. This study identified a significant increase in the incidence of grades II-IV aGVHD with major and minor ABO blood type mismatched between donor and recipient, which is identified as an independent risk factor, which aligns with previous research (45). ABO antigens are expressed on the surfaces of tissues like the gastrointestinal tract, liver, and skin, and they retain their original types due to the newly implanted hematopoietic system. Therefore, donor T lymphocytes will attack the incompatible antigens on tissue surfaces, leading to transplant rejection of corresponding organs. Hence, theoretically, HSCT patients with ABO blood type mismatched have a higher risk of GVHD occurrence. CYP2C19 enzyme is highly polymorphic, and its encoding gene is located on human chromosome 10. *CYP2C19* SNPs cause variations in enzyme activity, which affects the concentration of related drugs in the body, thereby affecting their efficacy and may lead to serious adverse reactions (46). The *CYP2C19* (99T>C) variants, known as CYP2C19*2, reduce the metabolic rate of drugs, including voriconazole and proton pump inhibitors. Following HSCT, cyclosporine is often co-administered with voriconazole or proton pump inhibitors, which can alter its concentration (47). Therefore, we speculate that *CYP2C19* (99T>C) variants may influence cyclosporine concentrations through drug interactions, thereby affecting aGVHD, which is an unexpected finding requiring further exploration and validation in subsequent studies.

Recent studies have identified that risk factors for EBV infection in patients after allo-HSCT, including the use of ATG, selective depletion of T cells, HLA mismatched, age ≥ 50 years old at the time of transplantation, and secondary transplantation (48). Since most patients lack fully HLA matched sibling donors, the main obstacles to using such donors for HSCT are graft rejection and GVHD (49–51). Strengthening pre-transplant conditioning regimens and T-cell depletion transplantation have reduced the incidence of these complications but may lead to slow immune reconstitution and increased rates of various infections. Therefore, we hypothesize that HLA mismatching increases the risk of EBV infection. In this study, we observed a significant reduction in EBV infection rates among recipients with HLA matches of 9/10-10/10. Similar to EBV infection, CMV reactivation is closely associated with the use of immunosuppressants. A previous study indicates that, in kidney transplant patients receiving immunosuppressive regimens with CsA, those with *CYP3A5* (219-237C>T) CC genotype exhibited higher dose-adjusted trough concentrations and dose-adjusted peak concentrations of CsA than those with variant genotypes. Consequently, patients with *CYP3A5* (219-237C>T) CC genotype may experience greater fluctuations in CsA dose adjustment to achieve target concentrations, thereby increasing the risk of CMV infection (25).

HC is a common complication of HSCT, with previous studies identifying factors as associated with its occurrence, including type of transplantation, age at transplantation, presence of GVHD, donor source, and the composition and intensity of the pre-transplant conditioning regimen (52–54). Consistent with prior literature, our study showed that male recipients and older children at the time of transplantation have a significantly higher incidence of HC (55). This could be attributed to estrogen’s protective roles in stabilizing microvasculature and the bladder mucosa, thereby increases the risk of HC in males. Moreover, older children experience higher rates of HC compared to younger children and are more likely to develop severe HC. This may be related to the less mature urinary and central nervous systems in younger children, which lead to more frequent urination, reducing the dwell time of drugs in the bladder and thereby minimizing the irritation and damage to the bladder mucosa by toxic metabolites, ultimately lowering the incidence of HC. Transporters such as the ATP-binding cassette family and the solute carrier family play key roles in drug transport and absorption. Additionally, this study identified wild-type *ABCB1* (1236C>T) and *SLCO1B1* (1865 + 4846T>C) as independent risk factors for developing HC.

Previous studies have found that CsA inhibits various influx and efflux transporters, including *ABCB1* and *SLCO1B1*, thereby increasing the area under the curve of other substrates such as bosentan, carprofen, and methotrexate. The degree of inhibition varies with different transporter polymorphisms, leading to variable increases in substrate concentrations (56–58). It was demonstrated that the increase in repaglinide concentration was 42% lower in subjects with the *SLCO1B1* (521T>C) variant genotype compared to those with the *SLCO1B1* (521T>C) TT genotype. This observation is due to the reduced function of OATP1B1 in individuals carrying the variant *SLCO1B1* c.521C allele (59). Therefore, we hypothesize that the wild-type *ABCB1* (1236C>T) and *SLCO1B1* (1865 + 4846T>C) alleles may exhibit higher activity than variant alleles. This could result in CsA significantly increasing the concentrations of other ABCB1 and SLCO1B1 substrates, including immunosuppressants (MTX, tacrolimus), leading to BK virus activation and subsequent HC. On the other hand, HC is influenced by various factors, often associated with high-dose pre-transplant chemotherapy toxicity, genetic polymorphisms in drug-metabolizing enzymes, viral infections, GVHD, patient age, gender, donor type, and transplantation method. Future studies should incorporate additional factors and expand sample sizes for further validation.

Furthermore, the present study identified that the *SLC29A1* (-162 + 228A>C) AC/CC genotype is an independent protective factor for both DFS and OS. SLC29A1 encodes human equilibrative nucleoside transporter 1 (hENT1) (60). As an equilibrative nucleoside transporter, hENT1 facilitates the influx of approximately 80% of cytarabine (Ara-C) into leukemia cells. A study involving 103 AML patients aged 16 to 76 years revealed that *SLC29A1* variants and haplotypes may influence the Ara-C uptake activity and the complete remission rate (60, 61). Wan et al. analyzed 19 *SLC29A1* SNPs in AML patients and found patients with low *SLC29A1* gene expression had shorter DFS and OS during Ara-C treatment. Moreover, significant differences were observed in the expression of the rs9394992 and rs324148 genotypes of *SLC29A1* gene between remission and relapse phases (62). The *SLC29A1* (-162 + 228A>C) variant, also known as rs693955, has been reported to be associated with a shorter duration of neutropenia following chemotherapy (63). However, no studies have yet demonstrated the relationship between this locus and prognosis. We hypothesize that the *SLC29A1* (-162 + 228A>C) variants may influence DFS and OS by affecting the hENT1 protein, thereby interfering with the absorption of cytarabine. In the future, we may verify this hypothesis by measuring plasma cytarabine levels using high-performance liquid chromatography.

However, our study has several limitations. First, the sample size is relatively small. Second, potential confounding factors, such as donor source variability, prior treatments, and differences in post-transplant care, could not be fully accounted for. Third, due to the retrospective nature of the study, external validation in independent cohorts and functional experimental confirmation are currently lacking. To address these issues, we will conduct a prospective, multicenter study with expanded sample size to validate our findings, construct clinically applicable predictive models, and establish mouse models of bone marrow transplantation harboring specific SNPs to investigate the pharmacokinetics and pharmacodynamics of CsA in the future.

In conclusion, our study has revealed correlations between CsA-related transporters and metabolic enzymes SNPs and post-transplant complications and prognosis, contributing to a better understanding of the interindividual difference in drug efficacy. Future studies on adjusting the dosage of drugs based on SNPs in clinical practice may be one of the options for improving the HSCT outcomes.

## Data Availability

The datasets presented in this study can be found in online repositories. The names of the repository/repositories and accession number(s) can be found below: https://db.cngb.org, CNP0004415, CNP0005143, CNP0007618.
