# Supplementary material for: Impact of cyclosporine A-related single nucleotide polymorphisms on post-transplant outcomes in pediatric hematologic malignancy patients undergoing allogeneic hematopoietic stem cell transplantation
Source: Front Immunol. 2025 Jul 22;16:1615976. doi: 10.3389/fimmu.2025.1615976 (PMC12321528; doi:10.3389/fimmu.2025.1615976)
Supplement: Supplementary file 1 [file Table1.docx]

Supplemental information for

Impact of cyclosporine A-related single nucleotide polymorphisms on post-transplant outcomes in pediatric hematologic malignancy patients undergoing allogeneic hematopoietic stem cell transplantation

**This file includes:**

Tables S1-S8

**Table S1.** Univariate and multivariate analyses of Peri-ES

| **Characteristics** | **Univariate analysis** | | **Multivariate analysis** | |
| --- | --- | --- | --- | --- |
|  | **HR (95% CI)** | ***P*-Value** | **HR (95% CI)** | ***P*-Value** |
| Gender, male vs. female | 1.04 (0.62 - 1.74) | 0.878 |  |  |
| Age, ≤ 104 months vs. > 104 months | 1.19 (0.75 - 1.89) | 0.449 |  |  |
| Weight, ≤ 26 kg vs. > 26 kg | 1.29 (0.81 - 2.05) | 0.261 |  |  |
| ABO match |  |  |  |  |
| Minor mismatched vs. Matched | 1.27 (0.70 - 2.31) | 0.400 |  |  |
| Major mismatched vs. Matched | **1.80 (0.92 - 3.52)** | **0.043** |  |  |
| Mismatched vs. Matched | 1.61 (0.61 - 4.25) | 0.238 |  |  |
| Donor type |  |  |  |  |
| MSD-HSCT vs. Haplo-HSCT | **0.22 (0.10 - 0.46)** | **0.014** | 0.51 (0.10 - 2.55) | 0.413 |
| MUD-HSCT vs. Haplo-HSCT | **0.47 (0.26 - 0.87)** | **0.043** | 1.26 (0.37 - 4.30) | 0.708 |
| UCB-HSCT vs. Haplo-HSCT | **1.90 (0.80 - 4.49)** | **0.049** | **2.82 (1.31 - 6.07)** | **0.008** |
| HLA match |  |  |  |  |
| 6/10-8/10 vs. 5/10 | 0.83 (0.50 - 1.39) | 0.452 | 0.68 (0.39 - 1.20) | 0.186 |
| 9/10-10/10 vs. 5/10 | **0.33 (0.19 - 0.60)** | **< 0.001** | **0.30 (0.11 - 0.83)** | **0.021** |
| BM state before HSCT, CR vs. PR/NR | 2.16 (0.95 - 4.93) | 0.159 |  |  |
| MNC, ≤ 6.83x10^8^/kg vs. > 6.83x10^8^/kg | 0.81 (0.51 - 1.29) | 0.356 |  |  |
| CD34+ cell, ≤ 6.59x10^6^/kg vs. > 6.59x10^6^/kg | 1.12 (0.70 - 1.78) | 0.616 |  |  |
| SNP |  |  |  |  |
| *ABCB1* (1000-44C>T) CT/TT vs. CC | 1.07 (0.67 - 1.71) | 0.767 |  |  |
| *ABCB1* (1236C>T) CT/TT vs. CC | 0.62 (0.28 - 1.38) | 0.130 |  |  |
| *ABCB1* (1554+24A>G) AG/GG vs. AA | 1.07 (0.67 - 1.71) | 0.767 |  |  |
| *ABCB1* (1725+38C>T) CT/TT vs. CC | 1.07 (0.67 - 1.71) | 0.767 |  |  |
| *ABCB1* (3435C>T) CT/TT vs. CC | 0.98 (0.61 - 1.59) | 0.941 |  |  |
| *BMP7* (g.57126159C>T) CT/TT vs. CC | **0.28 (0.13 - 0.61)** | **0.045** |  |  |
| *CYP2C19* (636G>A) GA/AA vs. GG | 0.80 (0.37 - 1.73) | 0.589 |  |  |
| *CYP2C19* (99T>C) TC/CC vs. TT | 0.99 (0.56 - 1.75) | 0.981 |  |  |
| *CYP2C8* (1291+106T>C) TC/CC vs. TT | 1.15 (0.57 - 2.31) | 0.692 |  |  |
| *CYP3A5* (219-237C>T) CT/TT vs. CC | 0.85 (0.53 - 1.35) | 0.465 |  |  |
| *MTHFR* (665C>T) CT/TT vs. CC | 0.80 (0.45 - 1.43) | 0.407 |  |  |
| *MTRR* (66A>G) AG/GG vs. AA | 1.20 (0.74 - 1.95) | 0.429 |  |  |
| *SLC29A1* (-162+228A>C) AC/CC vs. AA | 0.82 (0.33 - 2.03) | 0.623 |  |  |
| *SLCO1B1* (1865+4846T>C) TC/CC vs. TT | 1.14 (0.71 - 1.83) | 0.566 |  |  |
| *SLCO1B1* (521T>C) TC/CC vs. TT | 1.24 (0.66 - 2.31) | 0.453 |  |  |
| *UGT1A8* (518C>G) CG/GG vs. CC | 1.66 (0.99 - 2.77) | 0.072 |  |  |

Abbreviations: Peri-ES, peri-engraftment syndrome; HR, hazard ratio; CI, confidence interval; MSD, matched sibling donor; HSCT, hematopoietic stem cell transplantation; MUD, matched unrelated donor; UCB, umbilical cord blood; HLA, human leukocyte antigen; MNC, mononuclear cells; BM, bone marrow; PR, partial remission; NR, no remission; CR, complete remission; SNP, single nucleotide polymorphism.

**Table S2.** Univariate and multivariate analyses of II-IV aGVHD

| **Characteristics** | **Univariate analysis** | | **Multivariate analysis** | |
| --- | --- | --- | --- | --- |
|  | **HR (95% CI)** | ***P*-Value** | **HR (95% CI)** | ***P*-Value** |
| Gender, male vs. female | 1.12 (0.65 - 1.93) | 0.695 |  |  |
| Age, ≤ 104 months vs. > 104 months | 1.62 (0.98 - 2.67) | 0.059 |  |  |
| Weight, ≤ 26 kg vs. > 26 kg | **1.87 (1.13 - 3.09)** | **0.014** | **2.08 (1.22 - 3.58)** | **0.008** |
| ABO match |  |  |  |  |
| Minor mismatched vs. Matched | 1.96 (1.03 - 3.73) | 0.168 | **2.22 (1.18 - 4.17)** | **0.014** |
| Major mismatched vs. Matched | 2.11 (0.99 - 4.49) | 0.145 | **2.56 (1.28 - 5.13)** | **0.008** |
| Mismatched vs. Matched | 1.91 (0.62 - 5.89) | 0.913 | 1.83 (0.73 - 4.60) | 0.199 |
| Donor type |  |  |  |  |
| MSD-HSCT vs. Haplo-HSCT | 1.08 (0.23 - 5.03) | 0.724 |  |  |
| MUD-HSCT vs. Haplo-HSCT | 0 (0 - 0) | 0.737 |  |  |
| UCB-HSCT vs. Haplo-HSCT | **1.92 (0.39 - 9.37)** | **0.035** |  |  |
| HLA match |  |  |  |  |
| 6/10-8/10 vs. 5/10 | 0.85 (0.46 - 1.54) | 0.575 |  |  |
| 9/10-10/10 vs. 5/10 | 0.72 (0.39 - 1.32) | 0.281 |  |  |
| BM state before HSCT, CR vs. PR/NR | 1.16 (0.45 - 3.00) | 0.773 |  |  |
| MNC, ≤ 6.83x10^8^/kg vs. > 6.83x10^8^/kg | 0.79 (0.48 - 1.31) | 0.359 |  |  |
| CD34+ cell, ≤ 6.59x10^6^/kg vs. > 6.59x10^6^/kg | 0.90 (0.55 - 1.49) | 0.679 |  |  |
| SNP |  |  |  |  |
| *ABCB1* (1000-44C>T) CT/TT vs. CC | 0.75 (0.46 - 1.26) | 0.254 |  |  |
| *ABCB1* (1236C>T) CT/TT vs. CC | 1.33 (0.62 - 2.82) | 0.506 |  |  |
| *ABCB1* (1554+24A>G) AG/GG vs. AA | 0.75 (0.45 - 1.26) | 0.254 |  |  |
| *ABCB1* (1725+38C>T) CT/TT vs. CC | 0.75 (0.45 - 1.26) | 0.254 |  |  |
| *ABCB1* (3435C>T) CT/TT vs. CC | 0.99 (0.59 - 1.66) | 0.960 |  |  |
| *BMP7* (g.57126159C>T) CT/TT vs. CC | 0.49 (0.21 - 1.15) | 0.213 |  |  |
| *CYP2C19* (636G>A) GA/AA vs. GG | 1.17 (0.51 - 2.72) | 0.684 |  |  |
| *CYP2C19* (99T>C) TC/CC vs. TT | 1.64 (0.88 - 3.05) | 0.072 | **1.80 (1.02 - 3.17)** | **0.042** |
| *CYP2C8* (1291+106T>C) TC/CC vs. TT | 1.44 (0.69 - 2.99) | 0.388 |  |  |
| *CYP3A5* (219-237C>T) CT/TT vs. CC | 1.18 (0.71 - 1.96) | 0.505 |  |  |
| *MTHFR* (665C>T) CT/TT vs. CC | 0.87 (0.47 - 1.61) | 0.632 |  |  |
| *MTRR* (66A>G) AG/GG vs. AA | 1.51 (0.89 - 2.56) | 0.104 |  |  |
| *SLC29A1* (-162+228A>C) AC/CC vs. AA | 1.32 (0.54 - 3.25) | 0.587 |  |  |
| *SLCO1B1* (1865+4846T>C) TC/CC vs. TT | 1.15 (0.69 - 1.92) | 0.582 |  |  |
| *SLCO1B1* (521T>C) TC/CC vs. TT | 1.46 (0.75 - 2.86) | 0.201 |  |  |
| *UGT1A8* (518C>G) CG/GG vs. CC | 1.65 (0.95 - 2.87) | 0.112 |  |  |

Abbreviations: aGVHD, acute graft-versus-host disease; HR, hazard ratio; CI, confidence interval; MSD, matched sibling donor; HSCT, hematopoietic stem cell transplantation; MUD, matched unrelated donor; UCB, umbilical cord blood; HLA, human leukocyte antigen; MNC, mononuclear cells; BM, bone marrow; PR, partial remission; NR, no remission; CR, complete remission; SNP, single nucleotide polymorphism.

**Table S3.** Univariate and multivariate analyses of III-IV aGVHD

| **Characteristics** | **Univariate analysis** | | | **Multivariate analysis** | |
| --- | --- | --- | --- | --- | --- |
|  | **HR (95% CI)** | ***P-*value** | **HR (95% CI)** | | ***P-*value** |
| Gender, male vs. female | 1.09 (0.40 - 3.01) | 0.867 |  | |  |
| Age, ≤ 104 months vs. > 104 months | 2.09 (0.83 - 5.26) | 0.132 |  | |  |
| Weight, ≤ 26 kg vs. > 26 kg | 2.09 (0.83 - 5.26) | 0.132 |  | |  |
| ABO match |  |  |  | |  |
| Minor mismatched vs. Matched | 1.07 (0.30 - 3.81) | 0.921 |  | |  |
| Major mismatched vs. Matched | **2.96 (****0.89 - 9.90)** | **0.040** |  | |  |
| Mismatched vs. Matched | 0.94 (0.12 - 7.39) | 0.950 |  | |  |
| Donor type |  |  |  | |  |
| MSD-HSCT vs. Haplo-HSCT | 1.11 (0.24 - 5.21) | 0.891 |  | |  |
| MUD-HSCT vs. Haplo-HSCT | 0 (0 - 0) | 0.067 |  | |  |
| UCB-HSCT vs. Haplo-HSCT | 1.61 (0.37 - 7.07) | 0.453 |  | |  |
| HLA match |  |  |  | |  |
| 6/10-8/10 vs. 5/10 | 0.86 (0.26 - 2.79) | 0.797 |  | |  |
| 9/10-10/10 vs. 5/10 | 1.24 (0.42 - 3.67) | 0.702 |  | |  |
| BM state before HSCT, CR vs. PR/NR | Inf (Inf - Inf) | 0.225 |  | |  |
| MNC, ≤ 6.83x10^8^/kg vs. > 6.83x10^8^/kg | 0.58 (0.23 - 1.46) | 0.254 |  | |  |
| CD34+ cell, ≤ 6.59x10^6^/kg vs. > 6.59x10^6^/kg | 0.77 (0.31 - 1.94) | 0.578 |  | |  |
| SNP |  |  |  | |  |
| *ABCB1* (1000-44C>T) CT/TT vs. CC | 1.16 (0.45 - 2.95) | 0.763 |  | |  |
| *ABCB1* (1236C>T) CT/TT vs. CC | 2.23 (0.53 - 9.43) | 0.424 |  | |  |
| *ABCB1* (1554+24A>G) AG/GG vs. AA | 1.16 (0.45 - 2.95) | 0.763 |  | |  |
| *ABCB1* (1725+38C>T) CT/TT vs. CC | 1.16 (0.45 - 2.95) | 0.763 |  | |  |
| *ABCB1* (3435C>T) CT/TT vs. CC | 1.18 (0.45 - 3.06) | 0.743 |  | |  |
| *BMP7* (g.57126159C>T) CT/TT vs. CC | 0.67 (0.12 - 3.65) | 0.694 |  | |  |
| *CYP2C19* (636G>A) GA/AA vs. GG | 1.86 (0.39 - 8.89) | 0.316 |  | |  |
| CYP2C19 (99T>C) TC/CC vs. TT | 1.38 (0.45 - 4.24) | 0.536 |  | |  |
| *CYP2C8* (1291+106T>C) TC/CC vs. TT | 2.48 (0.62 - 9.92) | 0.360 |  | |  |
| *CYP3A5* (219-237C>T) CT/TT vs. CC | 1.84 (0.73 - 4.64) | 0.201 |  | |  |
| *MTHFR* (665C>T) CT/TT vs. CC | 0.42 (0.14 - 1.30) | 0.064 |  | |  |
| *MTRR* (66A>G) AG/GG vs. AA | 1.33 (0.51 - 3.44) | 0.550 |  | |  |
| *SLC29A1* (-162+228A>C) AC/CC vs. AA | Inf (Inf - Inf) | 0.199 |  | |  |
| *SLCO1B1* (1865+4846T>C) TC/CC vs. TT | 0.86 (0.34 - 2.20) | 0.746 |  | |  |
| *SLCO1B1* (521T>C) TC/CC vs. TT | 0.98 (0.29 - 3.38) | 0.979 |  | |  |
| UGT1A8 (518C>G) CG/GG vs. CC | 0.90 (0.31 - 2.59) | 0.838 |  | |  |

Abbreviations: aGVHD, acute graft-versus-host disease; HR, hazard ratio; CI, confidence interval; MSD, matched sibling donor; HSCT, hematopoietic stem cell transplantation; MUD, matched unrelated donor; UCB, umbilical cord blood; HLA, human leukocyte antigen; MNC, mononuclear cells; BM, bone marrow; PR, partial remission; NR, no remission; CR, complete remission; SNP, single nucleotide polymorphism. **Table S4.** Univariate and multivariate analyses of cGVHD

| **Characteristics** | **Univariate analysis** | | | **Multivariate analysis** | |
| --- | --- | --- | --- | --- | --- |
|  | **HR (95% CI)** | ***P*-value** | **HR (95% CI)** | | ***P-*value** |
| Gender, male vs. female | 0.90 (0.51 - 1.58) | 0.700 |  | |  |
| Age, ≤ 104 months vs. > 104 months | 0.88 (0.53 - 1.46) | 0.623 |  | |  |
| Weight, ≤ 26 kg vs. > 26 kg | 0.81 (0.49- 1.34) | 0.414 |  | |  |
| ABO match |  |  |  | |  |
| Minor mismatched vs. Matched | 0.63 (0.35 - 1.14) | 0.145 |  | |  |
| Major mismatched vs. Matched | 0.94 (0.49 - 1.81) | 0.851 |  | |  |
| Mismatched vs. Matched | 0.45 (0.19 - 1.08) | 0.170 |  | |  |
| Donor type |  |  |  | |  |
| MSD-HSCT vs. Haplo-HSCT | 0.65 (0.30 - 1.42) | 0.353 | 0.65 (0.26 - 1.63) | | 0.359 |
| MUD-HSCT vs. Haplo-HSCT | **0 (0-0)** | **< 0.001** | 0 (0 - inf) | | 0.947 |
| UCB-HSCT vs. Haplo-HSCT | 1.29 (0.57 - 2.94) | 0.502 | 1.29 (0.61 - 2.72) | | 0.512 |
| HLA match |  |  |  | |  |
| 6/10-8/10 vs. 5/10 | 1.17 (0.67 - 2.02) | 0.585 |  | |  |
| 9/10-10/10 vs. 5/10 | **0.34 (0.17 - 0.66)** | **0.002** |  | |  |
| BM state before HSCT, CR vs. PR/NR | 2.90 (1.20 - 6.98) | 0.120 |  | |  |
| MNC, ≤ 6.83x10^8^/kg vs. > 6.83x10^8^/kg | **0.49 (0.29 - 0.81)** | **0.005** |  | |  |
| CD34+ cell, ≤ 6.59x10^6^/kg vs. > 6.59x10^6^/kg | 1.15 (0.69 - 1.89) | 0.593 |  | |  |
| SNP |  |  |  | |  |
| *ABCB1* (1000-44C>T) CT/TT vs. CC | 1.10 (0.66 - 1.82) | 0.716 |  | |  |
| *ABCB1* (1236C>T) CT/TT vs. CC | 0.73 (0.32 - 1.69) | 0.405 |  | |  |
| *ABCB1* (1554+24A>G) AG/GG vs. AA | 1.10 (0.66 - 1.82) | 0.716 |  | |  |
| *ABCB1* (1725+38C>T) CT/TT vs. CC | 1.10 (0.66 - 1.82) | 0.716 |  | |  |
| *ABCB1* (3435C>T) CT/TT vs. CC | 0.94 (0.56 - 1.58) | 0.803 |  | |  |
| *BMP7* (g.57126159C>T) CT/TT vs. CC | 0.47 (0.20 - 1.08) | 0.185 |  | |  |
| *CYP2C19* (636G>A) GA/AA vs. GG | 1.49 (0.63 - 3.55) | 0.290 |  | |  |
| *CYP2C19* (99T>C) TC/CC vs. TT | 1.54 (0.82 - 2.88) | 0.128 |  | |  |
| *CYP2C8* (1291+106T>C) TC/CC vs. TT | 1.15 (0.57 - 2.33) | 0.711 |  | |  |
| *CYP3A5* (219-237C>T) CT/TT vs. CC | 0.92 (0.56 - 1.52) | 0.750 |  | |  |
| *MTHFR* (665C>T) CT/TT vs. CC | 0.98 (0.53 - 1.82) | 0.951 |  | |  |
| *MTRR* (66A>G) AG/GG vs. AA | 0.67 (0.40 - 1.11) | 0.133 |  | |  |
| *SLC29A1* (-162+228A>C) AC/CC vs. AA | 1.43 (0.60 - 3.42) | 0.483 |  | |  |
| *SLCO1B1* (1865+4846T>C) TC/CC vs. TT | 0.68 (0.41 - 1.14) | 0.129 |  | |  |
| *SLCO1B1* (521T>C) TC/CC vs. TT | **0.26 (0.14 - 0.49)** | **0.005** |  | |  |
| *UGT1A8* (518C>G) CG/GG vs. CC | 0.72 (0.40 - 1.30) | 0.239 |  | |  |

Abbreviations: cGVHD, chronic graft-versus-host disease; HR, hazard ratio; CI, confidence interval; MSD, matched sibling donor; HSCT, hematopoietic stem cell transplantation; MUD, matched unrelated donor; UCB, umbilical cord blood; HLA, human leukocyte antigen; MNC, mononuclear cells; BM, bone marrow; PR, partial remission; NR, no remission; CR, complete remission; SNP, single nucleotide polymorphism.

**Table S5.** Univariate and multivariate analyses of EBV infection

| **Characteristics** | **Univariate analysis** | | **Multivariate analysis** | |
| --- | --- | --- | --- | --- |
|  | **HR (95% CI)** | ***P*-value** | **HR (95% CI)** | ***P*-value** |
| Gender, male vs. female | 0.94 (0.56 - 1.57) | 0.804 |  |  |
| Age, ≤ 104 months vs. > 104 months | 0.76 (0.48 - 1.19) | 0.226 |  |  |
| Weight, ≤ 26 kg vs. > 26 kg | 1.09 (0.69 - 1.72) | 0.706 |  |  |
| ABO match |  |  |  |  |
| Minor mismatched vs. Matched | 1.25 (0.71 - 2.20) | 0.425 |  |  |
| Major mismatched vs. Matched | 1.30 (0.69 - 2.46) | 0.389 |  |  |
| Mismatched vs. Matched | 1.35 (0.54 - 3.37) | 0.465 |  |  |
| Donor type |  |  |  |  |
| MSD-HSCT vs. Haplo-HSCT | 0.70 (0.33 - 1.48) | 0.406 | 2.91 (0.91 - 9.31) | 0.710 |
| MUD-HSCT vs. Haplo-HSCT | 0.86 (0.46 - 1.60) | 0.638 | **5.22 (1.31 - 20.80)** | **0.019** |
| UCB-HSCT vs. Haplo-HSCT | **0.20 (0.10 - 0.40)** | **0.010** | 0.26 (0.06 - 1.13) | 0.072 |
| HLA match |  |  |  |  |
| 6/10-8/10 vs. 5/10 | 0.89 (0.53 - 1.50) | 0.659 | 0.81 (0.46 - 1.44) | 0.478 |
| 9/10-10/10 vs. 5/10 | **0.50 (0.28 - 0.89)** | **0.018** | **0.13 (0.04 - 0.49)** | **0.003** |
| BM state before HSCT, CR vs. PR/NR | 1.42 (0.60 - 3.37) | 0.488 |  |  |
| MNC, ≤ 6.83x10^8^/kg vs. > 6.83x10^8^/kg | 0.80 (0.51 - 1.27) | 0.342 |  |  |
| CD34+ cell, ≤ 6.59x10^6^/kg vs. > 6.59x10^6^/kg | 0.90 (0.57 - 1.42) | 0.644 |  |  |
| SNP |  |  |  |  |
| *ABCB1* (1000-44C>T) CT/TT vs. CC | **0.59 (0.37 - 0.95)** | **0.020** |  |  |
| *ABCB1* (1236C>T) CT/TT vs. CC | 1.24 (0.61 - 2.54) | 0.582 |  |  |
| *ABCB1* (1554+24A>G) AG/GG vs. AA | **0.59 (0.37 - 0.95)** | **0.020** |  |  |
| *ABCB1* (1725+38C>T) CT/TT vs. CC | **0.59 (0.37 - 0.95)** | **0.020** |  |  |
| *ABCB1* (3435C>T) CT/TT vs. CC | 1.35 (0.85 - 2.16) | 0.213 |  |  |
| *BMP7* (g.57126159C>T) CT/TT vs. CC | 0.55 (0.25 - 1.19) | 0.226 |  |  |
| *CYP2C19* (636G>A) GA/AA vs. GG | 0.83 (0.40 - 1.71) | 0.637 |  |  |
| *CYP2C19* (99T>C) TC/CC vs. TT | 1.13 (0.65 - 1.95) | 0.651 |  |  |
| *CYP2C8* (1291+106T>C) TC/CC vs. TT | 1.64 (0.86 - 3.13) | 0.202 |  |  |
| *CYP3A5* (219-237C>T) CT/TT vs. CC | **0.59 (0.37 - 0.92)** | **0.022** |  |  |
| *MTHFR* (665C>T) CT/TT vs. CC | 1.43 (0.84 - 2.42) | 0.220 |  |  |
| *MTRR* (66A>G) AG/GG vs. AA | 0.98 (0.61 - 1.57) | 0.931 |  |  |
| *SLC29A1* (-162+228A>C) AC/CC vs. AA | 0.67 (0.27 - 1.69) | 0.305 |  |  |
| *SLCO1B1* (1865+4846T>C) TC/CC vs. TT | 0.69 (0.43 - 1.11) | 0.107 |  |  |
| *SLCO1B1* (521T>C) TC/CC vs. TT | 0.72 (0.40 - 1.28) | 0.302 |  |  |
| *UGT1A8* (518C>G) CG/GG vs. CC | 0.91 (0.54 - 1.54) | 0.729 |  |  |

Abbreviations: EBV, Epstein-Barr virus; HR, hazard ratio; CI, confidence interval; MSD, matched sibling donor; HSCT, hematopoietic stem cell transplantation; MUD, matched unrelated donor; UCB, umbilical cord blood; HLA, human leukocyte antigen; MNC, mononuclear cells; BM, bone marrow; PR, partial remission; NR, no remission; CR, complete remission; SNP, single nucleotide polymorphism.

**Table S6.** Univariate and multivariate analyses of CMV infection

| **Characteristics** | **Univariate analysis** | | **Multivariate analysis** | |
| --- | --- | --- | --- | --- |
|  | **HR (95% CI)** | ***P*-value** | **HR (95% CI)** | ***P*-value** |
| Gender, male vs. female | 1.74 (1.05 - 2.89) | 0.052 |  |  |
| Age, ≤ 104 months vs. > 104 months | 0.82 (0.51 - 1.32) | 0.414 |  |  |
| Weight, ≤ 26 kg vs. > 26 kg | 0.73 (0.46 - 1.18) | 0.198 |  |  |
| ABO match |  |  |  |  |
| Minor mismatched vs. Matched | 1.34 (0.75 - 2.39) | 0.307 |  |  |
| Major mismatched vs. Matched | 0.98 (0.51 - 1.88) | 0.952 |  |  |
| Mismatched vs. Matched | 0.96 (0.38 - 2.44) | 0.928 |  |  |
| Donor type |  |  |  |  |
| MSD-HSCT vs. Haplo-HSCT | **0.38 (0.19 - 0.76)** | **0.046** |  |  |
| MUD-HSCT vs. Haplo-HSCT | 0.94 (0.51 - 1.74) | 0.847 |  |  |
| UCB-HSCT vs. Haplo-HSCT | 0.82 (0.39 - 1.70) | 0.612 |  |  |
| HLA match |  |  |  |  |
| 6/10-8/10 vs. 5/10 | 0.70 (0.41 - 1.21) | 0.194 |  |  |
| 9/10-10/10 vs. 5/10 | **0.44 (0.24 - 0.79)** | **0.006** |  |  |
| BM state before HSCT, CR vs. PR/NR | 1.06 (0.43 - 2.57) | 0.904 |  |  |
| MNC, ≤ 6.83x10^8^/kg vs. > 6.83x10^8^/kg | 1.14 (0.71 - 1.83) | 0.593 |  |  |
| CD34+ cell, ≤ 6.59x10^6^/kg vs. > 6.59x10^6^/kg | 1.24 (0.77 - 1.99) | 0.375 |  |  |
| SNP |  |  |  |  |
| *ABCB1* (1000-44C>T) CT/TT vs. CC | 1.07 (0.66 - 1.73) | 0.791 |  |  |
| *ABCB1* (1236C>T) CT/TT vs. CC | 1.46 (0.71 - 3.00) | 0.370 |  |  |
| *ABCB1* (1554+24A>G) AG/GG vs. AA | 1.07 (0.66 - 1.73) | 0.791 |  |  |
| *ABCB1* (1725+38C>T) CT/TT vs. CC | 1.07 (0.66 - 1.73) | 0.791 |  |  |
| *ABCB1* (3435C>T) CT/TT vs. CC | 0.90 (0.55 - 1.48) | 0.676 |  |  |
| *BMP7* (g.57126159C>T) CT/TT vs. CC | 1.01 (0.41 - 2.53) | 0.980 |  |  |
| *CYP2C19* (636G>A) GA/AA vs. GG | 1.04 (0.47 - 2.31) | 0.915 |  |  |
| *CYP2C19* (99T>C) TC/CC vs. TT | 0.72 (0.42 - 1.25) | 0.284 |  |  |
| *CYP2C8* (1291+106T>C) TC/CC vs. TT | **0.47 (0.21 - 1.08)** | **0.015** |  |  |
| *CYP3A5* (219-237C>T) CT/TT vs. CC | 0.70 (0.44 - 1.13) | 0.143 | **0.58 (0.36 - 0.93)** | **0.025** |
| *MTHFR* (665C>T) CT/TT vs. CC | 0.89 (0.50 - 1.59) | 0.674 |  |  |
| *MTRR* (66A>G) AG/GG vs. AA | 0.79 (0.49 - 1.28) | 0.346 |  |  |
| *SLC29A1* (-162+228A>C) AC/CC vs. AA | 1.11 (0.46 - 2.65) | 0.828 |  |  |
| *SLCO1B1* (1865+4846T>C) TC/CC vs. TT | 0.86 (0.53 - 1.40) | 0.529 |  |  |
| *SLCO1B1* (521T>C) TC/CC vs. TT | 1.35 (0.71 - 2.58) | 0.309 |  |  |
| *UGT1A8* (518C>G) CG/GG vs. CC | 0.81 (0.46 - 1.41) | 0.418 |  |  |

Abbreviations: CMV, cytomegalovirus; HR, hazard ratio; CI, confidence interval; MSD, matched sibling donor; HSCT, hematopoietic stem cell transplantation; MUD, matched unrelated donor; UCB, umbilical cord blood; HLA, human leukocyte antigen; MNC, mononuclear cells; BM, bone marrow; PR, partial remission; NR, no remission; CR, complete remission; SNP, single nucleotide polymorphism.

**Table S7.** Univariate and multivariate analyses of HC

| **Characteristics** | **Univariate analysis** | | **Multivariate analysis** | |
| --- | --- | --- | --- | --- |
|  | **HR (95% CI)** | ***P*-value** | **HR (95% CI)** | ***P*-value** |
| Gender, male vs. female | 2.02 (1.02 - 4.01) | 0.085 | **2.65 (1.14 - 6.17)** | **0.024** |
| Age, ≤ 104 months vs. > 104 months | **0.51 (0.27 - 0.96)** | **0.040** | **0.46 (0.24 - 0.90)** | **0.023** |
| Weight, ≤ 26 kg vs. > 26 kg | **0.52 (0.28 - 0.98)** | **0.047** |  |  |
| ABO match |  |  |  |  |
| Minor mismatched vs. Matched | 1.10 (0.52 - 2.35) | 0.801 |  |  |
| Major mismatched vs. Matched | 0.94 (0.40 - 2.21) | 0.881 |  |  |
| Mismatched vs. Matched | 0.62 (0.18 - 2.08) | 0.509 |  |  |
| Donor type |  |  |  |  |
| MSD-HSCT vs. Haplo-HSCT | 0.19 (0.07 - 0.52) | 0.068 |  |  |
| MUD-HSCT vs. Haplo-HSCT | 0.34 (0.15 - 0.77) | 0.061 |  |  |
| UCB-HSCT vs. Haplo-HSCT | 0.58 (0.22 - 1.51) | 0.355 |  |  |
| HLA match |  |  |  |  |
| 6/10-8/10 vs. 5/10 | 0.67 (0.34 - 1.41) | 0.313 |  |  |
| 9/10-10/10 vs. 5/10 | **0.36 (0.16 - 0.78)** | **0.014** |  |  |
| BM state before HSCT, CR vs. PR/NR | 0.89 (0.26 - 3.07) | 0.843 |  |  |
| MNC, ≤ 6.83x10^8^/kg vs. > 6.83x10^8^/kg | 0.64 (0.34 - 1.22) | 0.175 |  |  |
| CD34+ cell, ≤ 6.59x10^6^/kg vs. > 6.59x10^6^/kg | 1.04 (0.55 - 1.96) | 0.912 |  |  |
| SNP |  |  |  |  |
| *ABCB1* (1000-44C>T) CT/TT vs. CC | 1.28 (0.68 - 2.44) | 0.454 |  |  |
| *ABCB1* (1236C>T) CT/TT vs. CC | **0.45 (0.15 - 1.35)** | **0.047** | **0.39 (0.17 - 0.91)** | **0.030** |
| *ABCB1* (1554+24A>G) AG/GG vs. AA | 1.28 (0.68 - 2.44) | 0.454 |  |  |
| *ABCB1* (1725+38C>T) CT/TT vs. CC | 1.28 (0.68 - 2.44) | 0.454 |  |  |
| *ABCB1* (3435C>T) CT/TT vs. CC | 0.93 (0.48 - 1.81) | 0.828 |  |  |
| *BMP7* (g.57126159C>T) CT/TT vs. CC | 0.59 (0.19 - 1.84) | 0.463 |  |  |
| *CYP2C19* (636G>A) GA/AA vs. GG | 0.42 (0.16 - 1.14) | 0.221 |  |  |
| *CYP2C19* (99T>C) TC/CC vs. TT | 0.57 (0.27 - 1.18) | 0.192 |  |  |
| *CYP2C8* (1291+106T>C) TC/CC vs. TT | 0.73 (0.28 - 1.95) | 0.484 |  |  |
| *CYP3A5* (219-237C>T) CT/TT vs. CC | 0.63 (0.33 - 1.19) | 0.166 |  |  |
| *MTHFR* (665C>T) CT/TT vs. CC | 0.82 (0.39 - 1.76) | 0.597 |  |  |
| *MTRR* (66A>G) AG/GG vs. AA | 1.05 (0.55 - 2.02) | 0.878 |  |  |
| *SLC29A1* (-162+228A>C) AC/CC vs. AA | 1.68 (0.54 - 5.24) | 0.471 |  |  |
| *SLCO1B1* (1865+4846T>C) TC/CC vs. TT | **0.43 (0.22 - 0.82)** | **0.007** | **0.32 (0.16 - 0.63)** | **0.001** |
| *SLCO1B1* (521T>C) TC/CC vs. TT | 0.69 (0.30 - 1.59) | 0.440 |  |  |
| *UGT1A8* (518C>G) CG/GG vs. CC | 0.68 (0.32 - 1.44) | 0.266 |  |  |

Abbreviations: HC, hemorrhagic cystitis; HR, hazard ratio; CI, confidence interval; MSD, matched sibling donor; HSCT, hematopoietic stem cell transplantation; MUD, matched unrelated donor; UCB, umbilical cord blood; HLA, human leukocyte antigen; MNC, mononuclear cells; BM, bone marrow; PR, partial remission; NR, no remission; CR, complete remission; SNP, single nucleotide polymorphism.

**Table S8.** Univariate and multivariate analyses of CLS

| **Characteristics** | **Univariate analysis** | | **Multivariate analysis** | |
| --- | --- | --- | --- | --- |
|  | **HR (95% CI)** | ***P*-value** | **HR (95% CI)** | ***P*-value** |
| Gender, male vs. female | 1.36 (0.53 - 3.46) | 0.550 |  |  |
| Age, ≤ 104 months vs. > 104 months | 1.32 (0.56 - 3.11) | 0.524 |  |  |
| Weight, ≤ 26 kg vs. > 26 kg | 0.88 (0.38 - 2.08) | 0.774 |  |  |
| ABO match |  |  |  |  |
| Minor mismatched vs. Matched | 0.86 (0.30 - 2.46) | 0.784 |  |  |
| Major mismatched vs. Matched | 1.20 (0.40 - 3.64) | 0.740 |  |  |
| Mismatched vs. Matched | 0.57 (0.11 - 3.00) | 0.588 |  |  |
| Donor type |  |  |  |  |
| MSD-HSCT vs. Haplo-HSCT | 0.44 (0.10 - 1.89) | 0.415 |  |  |
| MUD-HSCT vs. Haplo-HSCT | 0.26 (0.08 - 0.86) | 0.154 |  |  |
| UCB-HSCT vs. Haplo-HSCT | 1.97 (0.49 - 7.90) | 0.218 |  |  |
| HLA match |  |  |  |  |
| 6/10-8/10 vs. 5/10 | 0.95 (0.39 - 2.33) | 0.907 | 0.95 (0.39 -2.33) | 0.906 |
| 9/10-10/10 vs. 5/10 | **0.19 (0.06 - 0.59)** | **0.016** | **0.19 (0.04 - 0.86)** | **0.031** |
| BM state before HSCT, CR vs. PR/NR | 1.54 (0.29 - 8.09) | 0.673 |  |  |
| MNC, ≤ 6.83x10^8^/kg vs. > 6.83x10^8^/kg | 0.70 (0.30 - 1.66) | 0.422 |  |  |
| CD34+ cell, ≤ 6.59x10^6^/kg vs. > 6.59x10^6^/kg | 2.08 (0.88 - 4.88) | 0.106 |  |  |
| SNP |  |  |  |  |
| *ABCB1* (1000-44C>T) CT/TT vs. CC | 0.96 (0.40 - 2.28) | 0.921 |  |  |
| *ABCB1* (1236C>T) CT/TT vs. CC | 0.73 (0.19 - 2.89) | 0.613 |  |  |
| *ABCB1* (1554+24A>G) AG/GG vs. AA | 0.96 (0.40 - 2.28) | 0.921 |  |  |
| *ABCB1* (1725+38C>T) CT/TT vs. CC | 0.96 (0.40 - 2.28) | 0.921 |  |  |
| *ABCB1* (3435C>T) CT/TT vs. CC | 0.61 (0.25 - 1.49) | 0.253 |  |  |
| *BMP7* (g.57126159C>T) CT/TT vs. CC | 1.28 (0.26 - 6.43) | 0.739 |  |  |
| *CYP2C19* (636G>A) GA/AA vs. GG | 0.90 (0.22 - 3.61) | 0.881 |  |  |
| *CYP2C19* (99T>C) TC/CC vs. TT | 2.21 (0.78 - 6.23) | 0.070 |  |  |
| *CYP2C8* (1291+106T>C) TC/CC vs. TT | 0.82 (0.22 - 3.06) | 0.751 |  |  |
| *CYP3A5* (219-237C>T) CT/TT vs. CC | 1.07 (0.45 - 2.52) | 0.880 |  |  |
| *MTHFR* (665C>T) CT/TT vs. CC | 0.57 (0.20 - 1.59) | 0.211 |  |  |
| *MTRR* (66A>G) AG/GG vs. AA | 0.81 (0.34 - 1.95) | 0.648 |  |  |
| *SLC29A1* (-162+228A>C) AC/CC vs. AA | 0.79 (0.16 - 3.93) | 0.745 |  |  |
| *SLCO1B1* (1865+4846T>C) TC/CC vs. TT | 1.79 (0.75 - 4.27) | 0.219 |  |  |
| *SLCO1B1* (521T>C) TC/CC vs. TT | 1.17 (0.37 - 3.68) | 0.777 |  |  |
| UGT1A8 (518C>G) CG/GG vs. CC | 1.08 (0.40 - 2.89) | 0.882 |  |  |

Abbreviations: HC, hemorrhagic cystitis; HR, hazard ratio; CI, confidence interval; MSD, matched sibling donor; HSCT, hematopoietic stem cell transplantation; MUD, matched unrelated donor; UCB, umbilical cord blood; HLA, human leukocyte antigen; MNC, mononuclear cells; BM, bone marrow; PR, partial remission; NR, no remission; CR, complete remission; SNP, single nucleotide polymorphism.
